# Analysis of Emergency Medical Response Team Performance during the International Winter Championships in Emergency Medicine

**DOI:** 10.3390/medicina58111578

**Published:** 2022-11-02

**Authors:** Michał Ćwiertnia, Tomasz Ilczak, Piotr Białoń, Arkadiusz Stasicki, Michał Szlagor, Mieczysław Dutka, Beata Kudłacik, Maciej B. Hajduga, Monika Mikulska, Rafał Bobiński, Marek Kawecki

**Affiliations:** 1 Department of Emergency Medicine, Faculty of Health Sciences, University of Bielsko-Biala, Willowa 2, 43-309 Bielsko-Biała, Poland; 2 Department of Biochemistry and Molecular Biology, Faculty of Health Sciences, University of Bielsko-Biala, Willowa 2, 43-309 Bielsko-Biała, Poland

**Keywords:** emergency medicine, cardiopulmonary resuscitation, advanced life support

## Abstract

*Background and Objectives*: Conducting advanced life support (ALS) according to the guidelines of the European Resuscitation Council (ERC) requires medical personnel to implement the appropriate emergency actions. In particular, these actions include chest compressions, airway management, artificial ventilation, defibrillation and the administering of medicines. An appropriate training system enables members of medical response teams (MRT) to acquire the essential knowledge and skills necessary to correctly conduct cardiopulmonary resuscitation (CPR). One way to improve the quality of interventions by MRT personnel is participation in emergency medicine championships. *Materials and Methods*: The research analysed assessment cards for tasks carried out during the International Winter Championships in Emergency Medicine in the years 2013–2020. The assessed tasks were prepared and led by European Resuscitation Council instructors of advanced life support. During ten-minute scenarios of simulated sudden cardiac arrest (SCA) in adults, the judges assessed the compliance of procedures with current ERC guidelines. This research analysed the performance of 309 teams from Poland made up of paramedics from medical response units from all over the country. *Results*: In most cases, the study showed significant differences in the percentage of correctly performed procedures between years. Most often, the highest percentage of correctly performed procedures was recorded in 2019 and 2020. The lowest percentage of correctly performed procedures was most often recorded in 2013. In subsequent years, the percentage of use of tracheal intubation decreased (from 54.76% to 31.25%) in favour of an increase in the use of supraglottic airway device SAD (from 35.71% to 59.38%). *Conclusions*: The research has shown that in subsequent years of the Championships, the quality of the majority of assessed procedures carried out by members of MRT gradually improved. The research authors also observed that in subsequent years, the percentage of intubations decreased in favour of SAD.

## 1. Introduction

In accordance with European Resuscitation Council guidelines, medical responses teams (MRT) should implement the appropriate emergency procedures during cardiopulmonary resuscitation (CPR). These procedures principally involve chest compressions, airway management, artificial ventilation, defibrillation, treatment of the reversible causes of sudden cardiac arrest (SCA), and administering drugs. Correct implementation of the advanced life support (ALS) algorithm requires medical response team members to have the appropriate knowledge and skills [1]. The training system is a valuable tool in improving the quality of CPR conducted by medical personnel [2,3,4]. The improvement of knowledge and skills is also helped by participation in contests, during which team members have the chance to ‘test themselves’ during simulated scenarios with patients in a life-threatening condition. Once such contests have finished, the results are a useful tool for acquiring information on the areas in which their knowledge and skills could be supplemented [5].

In Poland, the training of paramedics began in 1992. Initially, the professional qualification could only be gained by attending a two-year tertiary college. From the year 2000, paramedic training was also started on the basis of three-year bachelor’s studies [6]. In accordance with the State Emergency Medical Services Act, recruitment to two-year tertiary colleges was ended in 2013. The raising of the level of knowledge and skills among paramedics is conducted via training, in particular that certified by the European Resuscitation Council, as well as through participation in emergency medicine competitions. One such competition is the International Winter Championships in Emergency Medicine, which has been held since 2006 by the Emergency Medical Services in Bielsko-Biała. Teams participating in this competition are made up of three members from emergency medical response teams from Poland as well as teams invited from other European countries.

The aim of this paper is to assess the correctness of procedures implemented during simulated scenarios with adult patients during the International Winter Championships in Emergency Medicine in the years 2013–2020.

## 2. Materials and Methods

The research was conducted on the basis of analysis of Assessment Cards for tasks carried out during the International Winter Championships in Emergency Medicine in the years 2013–2020. In this period, 309 teams from Poland made up of paramedics from medical response units from all over the country took part in the Championships. The number of teams participating in the competition in individual years is as follows: 2013—37, 2014—45, 2015—43, 2016—42, 2017—37, 2018—40, 2019—31, 2020—34. Teams registered in advance by unit directors were able to participate in the competition. According to the regulations, people currently employed in given units could be team members. In each edition of the Championships, teams had to carry out 7 to 9 tasks. In this paper, assessment is made in each case of one of the individual tasks above, designed and conducted by a team of advanced life support instructors of European Resuscitation Council, employees of the Emergency Medical Service in Bielsko-Biała. These people were in no way connected to the teams participating in the competition. Teams from the Emergency Medical Services in Bielsko-Biała participated in the Championships but were not taken into account in the competition general classification. These teams were also excluded from participation in the research. The tasks analysed consisted of conducting CPR on an adult SCA patient during a simulated ten-minute scenario. To avoid the possibility that teams could predict the scenario, every year the tasks took place in different circumstances (a sports venue, a workplace, a shopping centre, etc.) in the town of Bielsko-Biała and the vicinity. During preparation of the annual assessment cards, the order of individual rhythms accompanying cardiac arrest were selected at random. The tasks were carried out using a Laerdal MegaCode Kelly mannequin. The assessment cards used by the judges to assess the teams were designed to assess the compliance of the procedures followed with ERC guidelines.

In the selected tasks, the judges assessed:

- The correct confirmation of SCA before attempt CPR.
- The duration of CPR cycles between successive heart rhythm analysis; for the purposes of the research, the assumed correct cycle duration was from 1:45 to 2:15 min.
- At what heart rhythm was the pulse checked or not checked.
- Defibrillation procedure—at what heart rhythm and with how energy was it conducted, how safe was it and what size paddles or electrodes were used.
- Route of administration, dose, earlier preparation and time when adrenaline was given.
- The manner of airway management and how artificial ventilation was conducted.
- The use of oxygen therapy.
- The use of capnography.

The above actions were taken into consideration in the research due to their crucial importance in the conducting of CPR in SCA patients.

The study project obtained a positive assessment from the Ethics Committee of the University of Bielsko-Biala (Decisions no. 2022/3/10E/11).

For statistical analysis of the results, the adopted level of significance was *p* = 0.05. For analysis of the quantitative variables presented using repeated measurements, a parametric test was used to compare the average values—ANOVA variance analysis with repeated measurements. To compare in detail the differences between the tested parameters in individual years, pairwise post hoc Tukey comparison tests were conducted. The calculations were made in the R statistical environment version 3.6.0, the PSPP program and MS Office 2019. The research results are presented as the percentage of a given emergency procedure correctly conducted by teams participating in the Championships in the years 2013–2020. As they did not demonstrate statistically significant differences to normal, assessment of the interdependencies between the quantitative variables were conducted using the Pearson correlation coefficient.

## 3. Results

The results after statistical analysis obtained during the research are presented in Table 1, Table 2, Table 3, Table 4, Table 5, Table 6, Table 7, Table 8, Table 9, Table 10, Table 11, Table 12, Table 13, Table 14, Table 15 and Table 16.

Table 1 presents the percentage of correctly conducted procedures carried out by MRT members during the Championships in the years 2013–2020.

Table 2 shows a comparison of confirmation of SCA (procedure A) by members of MRT in individual editions of the Championships.

The research showed statistically significant differences in confirmation of SCA before attempt CPR in individual years. This procedure was conducted correctly most frequently in the years 2015 (76.15%) and 2019 (76.09%). The lowest percentage of this procedure being conducted correctly was in 2016 (57.45%).

Table 3 compares the duration of CPR cycles (procedure B) between individual editions of the Championships.

The research showed statistically significant differences in duration of CPR cycles in individual years. The duration of cycles was most frequently correct in the years 2019 (60.87%) and 2020 (61.23%). In the years 2013 (49.69%), 2014 (48.96%) and 2016 (48.40%), the cycles duration was more frequently outside the correct range.

Table 4 compares pulse checking in the case of VT and PEA (procedure C) by MRT members between individual editions of the Championships.

The research showed statistically significant differences in checking the pulse at rhythms that could potentially give a pulse (VT and PEA) in individual years. Teams most frequently performed this procedure correctly by checking the pulse at these rhythms in 2014 (89.58%), 2015 (88.23%) and 2020 (87.95%). The lowest percentage of pulse checked at these rhythms was in 2013 (78.75%).

Table 5 compares no pulse checking in the case of ventricular fibrillation (VF) and asystole (procedure D) by MRT members between individual editions of the Championships.

The research showed statistically significant differences in no checking the pulse in the case of rhythms that could not give a pulse (VF and asystole) in individual years. Teams most frequently performed this procedure correctly by no checking the pulse in 2019 (92.03%) and 2020 (91.49%). In earlier years, this procedure was significantly more frequently performed incorrectly.

Table 6 compares defibrillation delivering in the case of VF and pVT (procedure E) by MRT members between individual editions of the Championships.

The research showed statistically significant differences in defibrillation delivering in the case of VF and pVT in individual years. Teams most frequently performed this procedure correctly by delivering defibrillation in the years 2017 (92.85%), 2019 (92.02%) and 2020 (93.48%). In earlier years, this procedure was significantly more frequently performed incorrectly.

Table 7 compares no delivering defibrillation in the case of PEA and asystole (procedure F) by MRT members between individual editions of the Championships.

The research showed statistically significant differences in no delivering defibrillation in the case of PEA and asystole in individual years. Teams most frequently performed this procedure correctly by no delivering defibrillation in 2019 (95.65%). This procedure was most frequently performed incorrectly in years 2013–2015.

Table 8 compares the energy used for defibrillation (procedure G) by MRT members between individual editions of the Championships.

The research showed statistically significant differences in the energy used for defibrillation in individual years. Defibrillation with the correct energy was conducted most frequently in the year 2020 (87.23%). The lowest percentage of defibrillation conducted with the correct energy was in 2013 (83.33%).

Table 9 compares the safety of defibrillation (procedure H) conducted by MRT members between individual editions of the Championships.

The research showed statistically significant differences in the safety of conducting defibrillation in individual years. Defibrillation was conducted in a safe manner most frequently in the years 2017 (91.83%) and 2020 (91.49%). The lowest percentage of defibrillation conducted in a safe manner was in the year 2015 (84.44%).

Table 10 compares the time of administering adrenaline (procedure J) by MRT members between individual editions of the Championships.

The research showed statistically significant differences in the administering of adrenaline in individual years. Adrenaline was administered at the correct time most frequently in the years 2018 (82.55%), 2019 (82.61%) and 2020 (80.65%). The lowest percentage of administering adrenaline at the correct time was in 2015 (44.68%).

Table 11 compares the earlier preparation of adrenaline (procedure K) by MRT members between individual editions of the Championships.

The research showed statistically significant differences in the earlier preparation of adrenaline in individual years. Teams acted correctly by preparing adrenaline earlier most frequently in the years 2019 (52.17%) and 2020 (51.06%). The lowest percentage of adrenaline prepared earlier was in the year 2013 (42.86%).

Table 12 compares the use of ventilation with oxygen (procedure N) by MRT members between individual editions of the Championships.

The research showed statistically significant differences in the frequency of ventilation with the use of oxygen in individual years. Teams acted correctly by using oxygen for ventilation most frequently in the years 2016 (97.87%) and 2019 (97.83%). The lowest percentage of ventilation with oxygen was in the year 2015 (94.12%).

Table 13 compares the use of capnography (procedure O) by MRT members between individual editions of the Championships.

The research showed statistically significant differences in the use of capnography in individual years. Teams acted correctly by using capnography most frequently in the years 2018 (79.07%), 2019 (78.26%) and 2020 (78.79%). The lowest percentage of use of capnography was in the years 2013 (38.09%) and 2014 (45.83%).

Table 14 compares the use of tracheal intubation (procedure P) by MRT members between individual editions of the Championships.

The research showed statistically significant differences in the use of tracheal intubation in individual years. Tracheal intubation was used most frequently in the years 2013 (54.76%) and 2015 (56.86%). The lowest percentage of tracheal intubation was in the years 2019 (32.61%) and 2020 (31.25%). 

Table 15 compares the use of SAD (procedure Q) by MRT members between individual editions of the Championships.

The research showed statistically significant differences in the use of SAD in individual years. SAD was used most frequently by teams in the years 2019 (60.84%) and 2020 (59.38%). The lowest percentage of the use of SAD was in the years 2013 (35.71%) and 2015 (31.37%).

The research also showed that in every edition of the Championships, all teams conducted part of the procedures entirely correctly. These procedures were the use of the correct size of paddle/electrode for defibrillation (procedure I) and administering the correct dose of adrenaline (procedure L) via the correct route (procedure M).

Analysis was conducted of the correlation between the individual procedures analysed. Table 16 presents the noted statistically significant correlation taking into account the degree of significance.

Statistically significant positive correlation was noted between the correct duration of CPR cycles and not delivering defibrillation in the case of PEA/asystole, early preparation of adrenaline, the use of SAD. A negative correlation was also shown between the correct duration of CPR cycles and the use of tracheal intubation. A statistically significant correlation was noted between not checking the pulse in the case of VF/asystole and defibrillation delivering in the case of VF/pVT, not delivering defibrillation in the case of PEA/asystole, the use of capnography, the use of SAD. There was also a negative correlation between not checking the pulse in the case of VF/asystole and the use of tracheal intubation. A significant positive correlation was noted between defibrillation delivering in the case of VF/pVT and not delivering defibrillation in the case of PEA/asystole, the use of defibrillation with the correct energy, earlier preparation of adrenaline, the use of capnography, the use of SAD. A negative correlation was also observed between the defibrillation delivering in the case of VF/pVT and the use of tracheal intubation. A positive correlation was found between not delivering defibrillation in the case of PEA/asystole and earlier preparation of adrenaline, the use of capnograph, the use of SAD. There was also a significant negative correlation between not delivering defibrillation in the case of PEA/asystole and the use of tracheal intubation. A significant positive correlation was noted between earlier preparation of adrenaline and the use of capnography, the use of SAD. A negative correlation was also observed between earlier preparation of adrenaline and the use of tracheal intubation. A significant positive correlation was noted between the use of capnography and the use of SAD. In addition, a negative correlation was shown between the use of capnography and the use of tracheal intubation. A very strong significant negative correlation was noted between the use of tracheal intubation and the use of SAD.

## 4. Discussion

CPR beginning should be preceded by correct confirmation of cardiac arrest [7,8,9]. According to ERC guidelines, medical response teams should confirm cardiac arrest in adults by simultaneous check of breathing and pulse. This procedure makes it possible to immediately begin CPR in SCA patients and minimises the risk of conducting CPR on patients with spontaneous circulation [1]. Our research has shown that in individual years, it was noted that cardiac arrest was not correctly confirmed in 23.85% to 42.55% of cases. Errors appeared most often in this area in the years 2013, 2014, 2016 and 2020. A certain additional difficulty for teams taking part in the Championships in the years 2016 and 2020 was that the scenario began with them taking over CPR from a bystander. In this situation, teams could have interpreted wrongly that confirmation of cardiac arrest was unnecessary, which resulted in mistakes being made.

ALS should be conducted in cycles, in which CPR is performed for 2 min between consecutive heart rhythm analysis [7,10]. For the research it was assumed that the correct duration of CPR cycles should be between 1:45 and 2:15 min. Analysis of the results showed that teams had difficulty to monitor the duration of CPR cycles during an intervention. In individual years, this error appeared in between 38.77% and 51,6% of cases. This may result from the fact that the leader of a three-person MRT must be engaged in manual intervention. Fernandez Castelao et al. [11] state that only by delegating tasks to individual team members can the leader maintain better CPR coordination. Our research showed a significant relation between conducting CPR in correct cycles and no delivering defibrillation in the case of PEA/asystole and earlier preparation of adrenaline. The research authors also noted that in comparison to earlier Championships, in the most recent editions there was a significant increase in the percentage of CPR cycles of correct duration.

During cardiopulmonary resuscitation, at successive heart rhythm analysis, check the pulse should be performed or omitted [1,7]. According to ERC guidelines, if the rhythm of the heart has visible QRS complexes, the pulse should be checked immediately. Thanks to this it is possible to detect return of spontaneous circulation (ROSC). If VF or asystole appears on the monitor, the pulse should not be checked. This procedure let minimise interruptions in chest compressions and increases the chances of survival for patients with SCA [1]. Our research has shown that in individual years, in the majority of cases, teams were able to identify when to check the pulse (from 78.75% to 89.58%) and when to omit checking for a pulse (from 84.53% to 92.03%). Statistical analysis showed that when MRTs did not check for a pulse in the case of VF and asystole, this had a significant relationship with the correct decision when to perform defibrillation.

During CPR, depending on the observed heart rhythm, defibrillation should either be delivered or not [12,13]. As numerous studies have shown [14,15,16], early use of defibrillation in patients with VF or pVT considerably improves the chances of survival for patients with SCA. Performing defibrillation on patients with other heart rhythms causes increased interruptions in chest compressions [12,13], and can result in damage to the heart muscle [17]. Our research has shown that in individual years, in the majority of cases, teams were able to successfully assess when defibrillation should be delivered (from 85.71% to 93.48%), and when it should not be delivered (from 89.59% to 95.65%). Statistical analysis showed that a higher percentage of defibrillation delivered by MRTs in the case of VF and pVT, had a significant relationship with not delivering defibrillation in the case of PEA and asystole. The most common procedural mistakes resulted from not delivering defibrillation in the case of VF or performing defibrillation in the case of asystole. The authors noticed that the percentage of mistakes saw a significant decrease in successive years, which may be the result of a better awareness of ERC guidelines.

During CPR on a cardiac arrest patient, subsequent defibrillation should be delivered at energy levels according to the recommendations of the device manufacturer [1]. Research [18,19] has shown that increasing the energy level in subsequent defibrillations increase the chances of a return of perfusing rhythm. Our research has shown that in individual years the percentage of defibrillation at the correct energy level was in the range from 83.33% to 87.23%. It was also observed that the considerable majority of defibrillations with incorrect energy levels took place at the second and third defibrillation. This situation mostly occurred when the team was using a defibrillator which did not automatically increase the energy level after defibrillation. The research results suggest that team members should pay more attention to the energy level they use to perform defibrillation. Devices that automatically increase the energy level after defibrillation could be useful in eliminating this type of error.

A key element during medical interventions provided by MRTs is team members ensuring that levels of safety are maintained. One moment when particular safety precautions should be taken is defibrillation [12,13]. Studies have described cases of patients or medical personnel suffering serious injuries during defibrillation [20,21,22]. Perkins et al. [11] describe how during defibrillation, care must be taken that no-one is touching the person undergoing defibrillation, and that oxygen equipment be moved to a safe distance. Our research has shown that in individual years, defibrillation was conducted in dangerous conditions in between 8.17% and 15.56% of cases. The majority of such cases involved oxygen equipment not being moved to a safe distance, which could result in burns to the patient and team members during treatment of the patient. Statistical analysis showed that safe defibrillation had a significant relationship to defibrillation being delivered in the case of VF and pVT. It is worth noting that in the most recent editions of the Championships, a significantly higher percentage of safe defibrillations was carried out than in the years from the beginning of the period studied.

According to ERC guidelines, adrenaline should be administered at appropriate moments during ALS [1]. Studies [23,24] have shown that early administration of adrenaline is related to improvement in the effectiveness of CPR and an increase in the survival rate of SCA patients. Our research has shown that in the years 2013–2016, the percentage of administration of adrenaline at the correct moment was between 44.68% and 71.43%. In the four subsequent years, this percentage was significantly higher, at between 77.55% and 82.61%. The years 2017–2020 also saw a significantly higher percentage of earlier preparation of adrenaline in relation to previous years. Improvement in the quality of procedures related to the administration of adrenaline could result from the fact that over the years, studies [23,24,25,26] have demonstrated an ever-increasing importance of administering adrenaline in cases of SCA. Equipped with this knowledge, teams may have paid ever more attention in subsequent years to the correct administration of adrenaline. Assessment of the administration of adrenaline also showed that MRTs have complete knowledge as to the dose and means of administering adrenaline. In every edition of the Championships, all teams administered adrenaline using the correct route and in the correct dose.

One element of CPR is conducting artificial ventilation. According to ERC guidelines, artificial ventilation should be conducted with the use of oxygen [12]. During the research, it was noted that the vast majority of teams taking part in every edition of the Championships correctly used oxygen for ventilation (from 94.12% to 97.87%). Soar et al. [1] state that this procedure aims to supply oxygen to the brain, thereby minimising hypoxic-ischemic damage to the central nervous system.

During CPR, members of MRTs should make use of capnography [12,13]. Monitoring end-tidal carbon dioxide levels makes it possible to monitor the quality of chest compressions [27], artificial ventilation [28], endotracheal tube position [29] and to identify any signs of ROSC [30]. Our research has shown that in subsequent editions of the Championships, teams made use of capnography significantly more frequently than in previous years. Statistical analysis showed that the use of capnography had a significant relationship with defibrillation delivering in the case of VF and pVT, not delivering defibrillation in the case of PEA/asystole, and the earlier preparation of adrenaline. The research results suggest that the more frequent use of capnography gives medical response teams greater control over procedures during CPR.

During CPR, medical response teams often conduct ventilation using advanced methods for airway management, such as tracheal intubation or SAD [31]. These methods make it possible to conduct asynchronous CPR [1,7], lowering the risk of gastric inflation [32], and enabling the use of capnography [29]. Our research has shown that the percentage of tracheal intubation used in subsequent editions of the Championships saw a considerable reduction. The research authors also noticed that in successive years, teams made use of SAD more and more often. Statistical analysis showed a highly significant correlation (*p* < 0.001), which confirmed the gradual decline in the number of tracheal intubations used in favour of more frequent use of SAD. This probably results from the fact that paramedics more often use SAD, according to ERC guidelines, as this does not require as much experience as tracheal intubation.

The quality of implemented emergency procedures has a direct influence on the effectiveness of CPR [1]. As shown in the scientific research, the quality of CPR can be influenced by the correct system for training medical personnel [2,3,4]. In the years the research was conducted, in Poland was a gradual increase in the percentage of paramedics having completed bachelor’s studies and those who had undergone ERC certified training. These factors could to a significant degree have influenced the improvement in the quality of procedures assessed during the research.

## 5. Limitations

The research authors are aware that one limitation is the fact that the research was conducted in simulated conditions. In the future, the research authors plan to conduct additional research in order to analyse the interventions of teams in actual working conditions with real patients.

## 6. Conclusions

Our research has demonstrated significant differences between individual years in the percentage of emergency procedures conducted correctly by paramedic teams. In the majority of cases, a gradual improvement was observed in the quality of procedures conducted. The research also showed a significant reduction in the use of intubation in favour of more frequent use of SAD.

## Figures and Tables

**Table 1:** Percentage of correctly conducted procedures carried out by MRT members during the Championships in the years 2013–2020.

	2013	2014	2015	2016	2017	2018	2019	2020
A	66.67%	68.75%	76.15%	57.45%	71.42%	74.42%	76.09%	68.75%
B	49.69%	48.96%	54.25%	48.40%	53.06%	51.16%	60.87%	61.23%
C	78.75%	89.58%	88.23%	82.97%	83.67%	86.04%	86.96%	87.95%
D	84.53%	85.42%	88.24%	90.43%	89.80%	90.70%	92.03%	91.49%
E	85.71%	89.58%	88.89%	89.36%	92.85%	90.70%	92.02%	93.48%
F	90.48%	89.59%	90.20%	91.49%	92.86%	93.02%	95.65%	93.62%
G	83.33%	85.41%	84.31%	85.11%	84.69%	86.04%	84.78%	87.23%
H	88.09%	87.50%	84.44%	87.23%	91.83%	90.70%	89.13%	91.49%
I	100%	100%	100%	100%	100%	100%	100%	100%
J	71.43%	68.75%	60.42%	44.68%	77.55%	82.55%	82.61%	80.65%
K	42.86%	47.91%	45.09%	44.68%	48.98%	48.84%	52.17%	51.06%
L	100%	100%	100%	100%	100%	100%	100%	100%
M	100%	100%	100%	100%	100%	100%	100%	100%
N	95.23%	95.83%	94.12%	97.87%	96.94%	95.35%	97.83%	95.74%
O	38.09%	45.83%	50.98%	57.45%	63.27%	79.07%	78.26%	78.79%
P	54.76%	52.08%	56.86%	48.93%	46.94%	41.86%	32.61%	31.25%
Q	35.71%	37.50%	31.37%	44.68%	44.90%	46.51%	60.84%	59.38%

A—correct confirmation of SCA; B—correct CPR cycles duration; C—pulse check in the case of ventricular tachycardia (VT) and pulseless electrical activity (PEA); D—no pulse check in the case of ventricular fibrillation (VF) and asystole; E—defibrillation deliver in the case of VF and pulseless ventricular tachycardia (pVT); F—defibrillation not deliver in the case of PEA and asystole; G—correct defibrillation energy; H—correct defibrillation safety; I—correct size of paddles/electrodes; J—correct time of adrenaline administration; K—adrenaline earlier preparation; L—correct adrenaline dose; M—correct route of administering adrenaline; N—ventilation using oxygen; O—use of capnography; P—use of tracheal intubation; Q—use of supraglottic airway device (SAD).

**Table 2:** Comparison of confirmation of SCA by members of MRT in individual editions of the Championships.

		2014	2015	2016	2017	2018	2019	2020
2013	MD	−2.08	−9.48	9.22	−4.75	−7.75	−9.42	−2.07
	*p*	*p* < 0.001	*p* < 0.001	*p* < 0.001	*p* < 0.001	*p* < 0.001	*p* < 0.001	*p* < 0.001
2014	MD	-	−7.40	11.30	−2.67	−5.67	−7.35	0.00
	*p*	-	*p* < 0.001	*p* < 0.001	*p* < 0.001	*p* < 0.001	*p* < 0.001	*p* = 1.000
2015	MD		-	18.70	4.73	1.73	0.05	7.40
	*p*		-	*p* < 0.001	*p* < 0.001	*p* < 0.001	*p* = 0.002	*p* < 0.001
2016	MD			-	−13.97	−16.97	−18.65	−11.30
	*p*			-	*p* < 0.001	*p* < 0.001	*p* < 0.001	*p* < 0.001
2017	MD				-	−3.00	−4.68	2.67
	*p*				-	*p* < 0.001	*p* < 0.001	*p* < 0.001
2018	MD					-	−1.68	5.67
	*p*					-	*p* < 0.001	*p* < 0.001
2019	MD						-	7.35
	*p*						-	*p* < 0.001

MD—mean difference; *p*—statistical significance.

**Table 3:** Comparison of the duration of CPR cycles between individual editions of the Championships.

		2014	2015	2016	2017	2018	2019	2020
2013	MD	0.74	−4.55	1.30	−3.36	−1.46	−11.18	−11.53
	*p*	*p* < 0.001	*p* < 0.001	*p* < 0.001	*p* < 0.001	*p* < 0.001	*p* < 0.001	*p* < 0.001
2014	MD	-	−5.29	0.56	−4.10	−2.20	−11.92	−12.27
	*p*	-	*p* < 0.001	*p* < 0.001	*p* < 0.001	*p* < 0.001	*p* < 0.001	*p* < 0.001
2015	MD		-	5.85	1.19	3.09	−6.63	−6.98
	*p*		-	*p* < 0.001	*p* < 0.001	*p* < 0.001	*p* < 0.001	*p* < 0.001
2016	MD			-	−4.66	−2.76	−12.48	−12.83
	*p*			-	*p* < 0.001	*p* < 0.001	*p* < 0.001	*p* < 0.001
2017	MD				-	1.90	−7.82	−8.17
	*p*				-	*p* < 0.001	*p* < 0.001	*p* < 0.001
2018	MD					-	−9.72	−10.07
	*p*					-	*p* < 0.001	*p* < 0.001
2019	MD						-	−0.35
	*p*						-	*p* < 0.001

MD—mean difference; *p*—statistical significance.

**Table 4:** Comparison of pulse checking in the case of VT and PEA by MRT members between individual editions of the Championships.

		2014	2015	2016	2017	2018	2019	2020
2013	MD	−10.82	−9.47	−4.21	−4.91	−7.28	−8.21	−9.19
	*p*	*p* < 0.001	*p* < 0.001	*p* < 0.001	*p* < 0.001	*p* < 0.001	*p* < 0.001	*p* < 0.001
2014	MD	-	1.35	6.61	5.91	3.54	2.61	1.63
	*p*	-	*p* < 0.001	*p* < 0.001	*p* < 0.001	*p* < 0.001	*p* < 0.001	*p* < 0.001
2015	MD		-	5.26	4.56	2.19	1.26	0.28
	*p*		-	*p* < 0.001	*p* < 0.001	*p* < 0.001	*p* < 0.001	*p* < 0.001
2016	MD			-	−0.70	−3.07	−4.00	−4.98
	*p*			-	*p* < 0.001	*p* < 0.001	*p* < 0.001	*p* < 0.001
2017	MD				-	−2.37	−3.30	−4.28
	*p*				-	*p* < 0.001	*p* < 0.001	*p* < 0.001
2018	MD					-	−0.93	−1.91
	*p*					-	*p* < 0.001	*p* < 0.001
2019	MD						-	−0.98
	*p*						-	*p* < 0.001

MD—mean difference; *p*—statistical significance.

**Table 5:** Comparison of no pulse checking in the case of VF and asystole between individual editions of the Championships.

		2014	2015	2016	2017	2018	2019	2020
2013	MD	−0.88	−3.70	−5.89	−5.26	−6.16	−7.50	−6.95
	*p*	*p* < 0.001	*p* < 0.001	*p* < 0.001	*p* < 0.001	*p* < 0.001	*p* < 0.001	*p* < 0.001
2014	MD	-	−2.82	−5.01	−4.38	−5.28	−6.62	−6.07
	*p*	-	*p* < 0.001	*p* < 0.001	*p* < 0.001	*p* < 0.001	*p* < 0.001	*p* < 0.001
2015	MD		-	−2.19	−1.56	−2.46	−3.80	−3.25
	*p*		-	*p* < 0.001	*p* < 0.001	*p* < 0.001	*p* < 0.001	*p* < 0.001
2016	MD			-	0.63	−0.27	−1.61	−1.06
	*p*			-	*p* < 0.001	*p* < 0.001	*p* < 0.001	*p* < 0.001
2017	MD				-	−0.90	−2.24	−1.69
	*p*				-	*p* < 0.001	*p* < 0.001	*p* < 0.001
2018	MD					-	−1.34	−0.79
	*p*					-	*p* < 0.001	*p* < 0.001
2019	MD						-	0.55
	*p*						-	*p* < 0.001

MD—mean difference; *p*—statistical significance.

**Table 6:** Comparison of defibrillation delivering in the case of VF and pVT between individual editions of the Championships.

		2014	2015	2016	2017	2018	2019	2020
2013	MD	−3.86	−3.17	−3.64	−7.13	−4.98	6.31	−7.76
	*p*	*p* < 0.001	*p* < 0.001	*p* < 0.001	*p* < 0.001	*p* < 0.001	*p* < 0.001	*p* < 0.001
2014	MD	-	0.69	0.22	−3.27	−1.12	−2.45	−3.90
	*p*	-	*p* < 0.001	*p* < 0.001	*p* < 0.001	*p* < 0.001	*p* < 0.001	*p* < 0.001
2015	MD		-	−0.47	−3.96	−1.81	−3.14	−4.59
	*p*		-	*p* < 0.001	*p* < 0.001	*p* < 0.001	*p* < 0.001	*p* < 0.001
2016	MD			-	−3.49	−1.34	−2.67	−4.12
	*p*			-	*p* < 0.001	*p* < 0.001	*p* < 0.001	*p* < 0.001
2017	MD				-	2.15	0.82	−0.63
	*p*				-	*p* < 0.001	*p* < 0.001	*p* < 0.001
2018	MD					-	−1.33	−2.78
	*p*					-	*p* < 0.001	*p* < 0.001
2019	MD						-	−1.45
	*p*						-	*p* < 0.001

MD—mean difference; *p*—statistical significance.

**Table 7:** Comparison of no delivering defibrillation in the case of PEA and asystole between individual editions of the Championships.

		2014	2015	2016	2017	2018	2019	2020
2013	MD	0.90	0.29	−1.00	−2.37	−2.53	−5.17	−3.13
	*p*	*p* < 0.001	*p* < 0.001	*p* < 0.001	*p* < 0.001	*p* < 0.001	*p* < 0.001	*p* < 0.001
2014	MD	-	−0.61	−1.90	−3.27	−3.43	−6.07	−4.03
	*p*	-	*p* < 0.001	*p* < 0.001	*p* < 0.001	*p* < 0.001	*p* < 0.001	*p* < 0.001
2015	MD		-	−1.29	−2.66	−2.82	−5.46	−3.42
	*p*		-	*p* < 0.001	*p* < 0.001	*p* < 0.001	*p* < 0.001	*p* < 0.001
2016	MD			-	−1.37	−1.53	−4.17	−2.13
	*p*			-	*p* < 0.001	*p* < 0.001	*p* < 0.001	*p* < 0.001
2017	MD				-	−0.16	−2.80	−0.76
	*p*				-	*p* < 0.001	*p* < 0.001	*p* < 0.001
2018	MD					-	−2.64	−0.60
	*p*					-	*p* < 0.001	*p* < 0.001
2019	MD						-	2.04
	*p*						-	*p* < 0.001

MD—mean difference; *p*—statistical significance.

**Table 8:** Comparison of the energy use for defibrillation between individual editions of the Championships.

		2014	2015	2016	2017	2018	2019	2020
2013	MD	−2.07	−0.97	−1.77	−1.35	−2.70	−1.45	−3.89
	*p*	*p* < 0.001	*p* < 0.001	*p* < 0.001	*p* < 0.001	*p* < 0.001	*p* < 0.001	*p* < 0.001
2014	MD	-	1.10	0.30	0.72	−0.63	0.62	−1.82
	*p*	-	*p* < 0.001	*p* < 0.001	*p* < 0.001	*p* < 0.001	*p* < 0.001	*p* < 0.001
2015	MD		-	−0.80	−0.38	−1.73	−0.48	−2.92
	*p*		-	*p* < 0.001	*p* < 0.001	*p* < 0.001	*p* < 0.001	*p* < 0.001
2016	MD			-	0.42	−0.93	0.32	−2.12
	*p*			-	*p* < 0.001	*p* < 0.001	*p* < 0.001	*p* < 0.001
2017	MD				-	−1.35	−0.10	−2.54
	*p*				-	*p* < 0.001	*p* < 0.001	*p* < 0.001
2018	MD					-	1.25	−1.19
	*p*					-	*p* < 0.001	*p* < 0.001
2019	MD						-	−2.44
	*p*						-	*p* < 0.001

MD—mean difference; *p*—statistical significance.

**Table 9:** Comparison of the safety of defibrillation between individual editions of the Championships.

		2014	2015	2016	2017	2018	2019	2020
2013	MD	0.60	0.60	0.87	−3.73	−2.60	−1.04	−3.39
	*p*	*p* < 0.001	*p* < 0.001	*p* < 0.001	*p* < 0.001	*p* < 0.001	*p* < 0.001	*p* < 0.001
2014	MD	-	3.06	0.27	−4.33	−3.20	−1.64	−3.99
	*p*	-	*p* < 0.001	*p* < 0.001	*p* < 0.001	*p* < 0.001	*p* < 0.001	*p* < 0.001
2015	MD		-	−2.79	−7.39	−6.26	−4.70	−7.05
	*p*		-	*p* < 0.001	*p* < 0.001	*p* < 0.001	*p* < 0.001	*p* < 0.001
2016	MD			-	−4.60	−3.47	−1.91	−4.26
	*p*			-	*p* < 0.001	*p* < 0.001	*p* < 0.001	*p* < 0.001
2017	MD				-	−1.13	2.69	0.34
	*p*				-	*p* < 0.001	*p* < 0.001	*p* < 0.001
2018	MD					-	1.56	−0.79
	*p*					-	*p* < 0.001	*p* < 0.001
2019	MD						-	−2.35
	*p*						-	*p* < 0.001

MD—mean difference; *p*—statistical significance.

**Table 10:** Comparison of administering adrenaline at the correct time between individual editions of the Championships.

		2014	2015	2016	2017	2018	2019	2020
2013	MD	2.69	11.02	26.76	−6.11	−11.11	−11.18	−9.21
	*p*	*p* < 0.001	*p* < 0.001	*p* < 0.001	*p* < 0.001	*p* < 0.001	*p* < 0.001	*p* < 0.001
2014	MD	-	8.33	24.07	−8.80	−13.80	−13.87	−11.90
	*p*	-	*p* < 0.001	*p* < 0.001	*p* < 0.001	*p* < 0.001	*p* < 0.001	*p* < 0.001
2015	MD		-	15.74	−17.13	−22.13	−22.20	−20.23
	*p*		-	*p* < 0.001	*p* < 0.001	*p* < 0.001	*p* < 0.001	*p* < 0.001
2016	MD			-	−32.87	−37.87	−37.94	−35.97
	*p*			-	*p* < 0.001	*p* < 0.001	*p* < 0.001	*p* < 0.001
2017	MD				-	−5.00	−5.07	−3.10
	*p*				-	*p* < 0.001	*p* < 0.001	*p* < 0.001
2018	MD					-	−0.07	1.90
	*p*					-	*p* < 0.001	*p* < 0.001
2019	MD						-	1.97
	*p*						-	*p* < 0.001

MD—mean difference; *p*—statistical significance.

**Table 11:** Comparison of the earlier preparation of adrenaline between individual editions of the Championships.

		2014	2015	2016	2017	2018	2019	2020
2013	MD	−5.04	−2.22	−1.81	−6.11	−5.97	−9.31	−8.19
	*p*	*p* < 0.001	*p* < 0.001	*p* < 0.001	*p* < 0.001	*p* < 0.001	*p* < 0.001	*p* < 0.001
2014	MD	-	2.82	3.23	−1.07	−0.93	−4.27	−3.15
	*p*	-	*p* < 0.001	*p* < 0.001	*p* < 0.001	*p* < 0.001	*p* < 0.001	*p* < 0.001
2015	MD		-	0.41	−3.89	−3.75	−7.09	−5.97
	*p*		-	*p* < 0.001	*p* < 0.001	*p* < 0.001	*p* < 0.001	*p* < 0.001
2016	MD			-	−4.30	−4.16	−7.50	−6.38
	*p*			-	*p* < 0.001	*p* < 0.001	*p* < 0.001	*p* < 0.001
2017	MD				-	0.14	−3.20	−2.08
	*p*				-	*p* < 0.001	*p* < 0.001	*p* < 0.001
2018	MD					-	−3.34	−2.22
	*p*					-	*p* < 0.001	*p* < 0.001
2019	MD						-	1.12
	*p*						-	*p* < 0.001

MD—mean difference; *p*—statistical significance.

**Table 12:** Comparison of the use of ventilation with oxygen between individual editions of the Championships.

		2014	2015	2016	2017	2018	2019	2020
2013	MD	−0.59	1.12	−2.63	−1.70	−0.11	−2.60	−0.50
	*p*	*p* < 0.001	*p* < 0.001	*p* < 0.001	*p* < 0.001	*p* < 0.001	*p* < 0.001	*p* < 0.001
2014	MD	-	1.71	−2.04	−1.11	0.48	−2.01	0.09
	*p*	-	*p* < 0.001	*p* < 0.001	*p* < 0.001	*p* < 0.001	*p* < 0.001	*p* < 0.001
2015	MD		-	−3.75	−2.82	−1.23	−3.72	−1.62
	*p*		-	*p* < 0.001	*p* < 0.001	*p* < 0.001	*p* < 0.001	*p* < 0.001
2016	MD			-	0.93	2.52	0.03	2.13
	*p*			-	*p* < 0.001	*p* < 0.001	*p* < 0.001	*p* < 0.001
2017	MD				-	1.59	−0.90	1.20
	*p*				-	*p* < 0.001	*p* < 0.001	*p* < 0.001
2018	MD					-	−2.49	−0.39
	*p*					-	*p* < 0.001	*p* < 0.001
2019	MD						-	2.10
	*p*						-	*p* < 0.001

MD—mean difference; *p*—statistical significance.

**Table 13:** Comparison of the use of capnography between individual editions of the Championships.

		2014	2015	2016	2017	2018	2019	2020
2013	MD	−7.73	−12.88	−19.35	−25.17	−40.97	−40.17	−40.69
	*p*	*p* < 0.001	*p* < 0.001	*p* < 0.001	*p* < 0.001	*p* < 0.001	*p* < 0.001	*p* < 0.001
2014	MD	-	−5.15	−11.62	−17.44	−33.24	−32.44	−32.96
	*p*	-	*p* < 0.001	*p* < 0.001	*p* < 0.001	*p* < 0.001	*p* < 0.001	*p* < 0.001
2015	MD		-	−6.47	−12.29	−28.09	−27.29	−27.81
	*p*		-	*p* < 0.001	*p* < 0.001	*p* < 0.001	*p* < 0.001	*p* < 0.001
2016	MD			-	−5.82	−21.62	−20.82	−21.34
	*p*			-	*p* < 0.001	*p* < 0.001	*p* < 0.001	*p* < 0.001
2017	MD				-	−15.80	−15.00	−15.52
	*p*				-	*p* < 0.001	*p* < 0.001	*p* < 0.001
2018	MD					-	0.80	0.28
	*p*					-	*p* < 0.001	*p* < 0.001
2019	MD						-	−0.52
	*p*						-	*p* < 0.001

MD—mean difference; *p*—statistical significance.

**Table 14:** Comparison of the use of tracheal intubation between individual editions of the Championships.

		2014	2015	2016	2017	2018	2019	2020
2013	MD	2.69	−2.09	5.84	7.83	12.91	22.15	23.52
	*p*	*p* < 0.001	*p* < 0.001	*p* < 0.001	*p* < 0.001	*p* < 0.001	*p* < 0.001	*p* < 0.001
2014	MD	-	−4.78	3.15	5.14	10.22	19.46	20.83
	*p*	-	*p* < 0.001	*p* < 0.001	*p* < 0.001	*p* < 0.001	*p* < 0.001	*p* < 0.001
2015	MD		-	7.93	9.92	15.00	24.24	25.61
	*p*		-	*p* < 0.001	*p* < 0.001	*p* < 0.001	*p* < 0.001	*p* < 0.001
2016	MD			-	1.99	7.07	16.31	17.68
	*p*			-	*p* < 0.001	*p* < 0.001	*p* < 0.001	*p* < 0.001
2017	MD				-	5.08	14.32	15.69
	*p*				-	*p* < 0.001	*p* < 0.001	*p* < 0.001
2018	MD					-	9.24	10.61
	*p*					-	*p* < 0.001	*p* < 0.001
2019	MD						-	1.37
	*p*						-	*p* < 0.001

MD—mean difference; *p*—statistical significance.

**Table 15:** Comparison of the use of SAD between individual editions of the Championships.

		2014	2015	2016	2017	2018	2019	2020
2013	MD	−1.78	4.35	−8.96	−9.18	−10.79	−25.13	−23.66
	*p*	*p* < 0.001	*p* < 0.001	*p* < 0.001	*p* < 0.001	*p* < 0.001	*p* < 0.001	*p* < 0.001
2014	MD	-	6.13	−7.18	−7.40	−9.01	−23.35	−21.88
	*p*	-	*p* < 0.001	*p* < 0.001	*p* < 0.001	*p* < 0.001	*p* < 0.001	*p* < 0.001
2015	MD		-	−13.31	−13.53	−15.14	−29.48	−28.01
	*p*		-	*p* < 0.001	*p* < 0.001	*p* < 0.001	*p* < 0.001	*p* < 0.001
2016	MD			-	−0.22	−1.83	−16.17	−14.70
	*p*			-	*p* < 0.001	*p* < 0.001	*p* < 0.001	*p* < 0.001
2017	MD				-	−1.61	−15.95	−14.48
	*p*				-	*p* < 0.001	*p* < 0.001	*p* < 0.001
2018	MD					-	−14.34	−12.87
	*p*					-	*p* < 0.001	*p* < 0.001
2019	MD						-	1.47
	*p*						-	*p* < 0.001

MD—mean difference; *p*—statistical significance.

**Table 16:** ‘R’ correlation coefficient significance for procedures whose correctness saw a significant change in the years 2013–2020.

		B	D	E	F	H	K	O	P
D	r	0.63	-						
	*p*	0.093	-						
E	r	0.67	0.79 *	-					
	*p*	0.071	0.020	-					
F	r	0.75 *	0.84 **	0.72 *	-				
	*p*	0.032	0.009	0.046	-				
H	r	0.40	0.57	0.71 *	0.37	-			
	*p*	0.322	0.139	0.049	0.362	-			
K	r	0.75 *	0.69	0.89 **	0.80 *	0.64	-		
	*p*	0.032	0.060	0.003	0.017	0.088	-		
O	r	0.68	0.92	0.92 **	0.88 **	0.70	0.84 **	-	
	*p*	0.065	0.001	0.001	0.004	0.053	0.010	-	
P	r	−0.79 *	−0.79 *	−0.78 *	−0.90 **	−0.68	−0.87 **	−0.90 **	-
	*p*	0.020	0.020	0.024	0.002	0.065	0.005	0.003	-
Q	r	0.75 *	0.80 *	0.75 *	0.92 *	0.60	0.83	0.85 **	*** −0.98
	*p*	0.031	0.018	0.030	0.001	0.117	0.010	0.007	<0.001

r—Pearson correlation coefficient, *p*—statistical significance, * *p* < 0.05; ** *p* < 0.01; *** *p* < 0.001.

## Data Availability

The data presented in this study are available on request from the corresponding author.

## Abbreviations

ALS	advanced life support
ERC	European Resuscitation Council
MRT	medical response team
CPR	cardiopulmonary resuscitation
SCA	sudden cardiac arrest
SAD	supraglottic airway device
VT	ventricular tachycardia
PEA	pulseless electrical activity
VF	ventricular fibrillation
pVT	pulseless ventricular tachycardia
ROSC	return of spontaneous circulation

## References

[1] Soar J., Bottiger B.W., Carli P., Couper K., Deakin C.D., Djarv T., Lott C., Olasveengen T., Paal P., Pellis T. (2021). European Resuscitation Council Guidelines 2021: Adult advanced life support. Resuscitation.

[2] Crowe C., Bobrow B.J., Vadeboncoeur T.F., Dameff C., Stolz U., Silver A., Roosa J., Page R., LoVecchio F., Spaite D.W. (2015). Measuring and improving cardiopulmonary resuscitation quality inside the emergency department. Resuscitation.

[3] Laco R.B., Stuart W.P. (2021). Simulation-Based Training Program to Improve Cardiopulmonary Resuscitation and Teamwork Skills for the Urgent Care Clinic Staff. Mil. Med..

[4] Young A.K., Maniaci M.J., Simon L.V., Lowman P.E., McKenna R.T., Thomas C.S., Cochuyt J.J., Vadeboncoeur T.F. (2020). Use of a simulation-based advanced resuscitation training curriculum: Impact on cardiopulmonary resuscitation quality and patient outcomes. J. Intensive Care Soc..

[5] Smart J.R., Kranz K., Carmona F., Lindner T.W., Newton A. (2015). Does real-time objective feedback and competition improve performance and quality in manikin CPR training—A prospective observational study from several European *EMS*. Scand. J. Trauma Resusc. Emerg. Med..

[6] Sip M., Juskowiak K., Zgorzalewicz-Stachowiak M., Zeńczak-Praga K., Rybakowski M., Podlewski R. (2019). Evolution of paramedic profession in Poland and jobrelated risk. Hygeia Public Health.

[7] Panchal A.R., Bartos J.A., Cabanas J.G., Donnino M.W., Drennan I.R., Hirsch K.G., Kudenchuk P.J., Kurz M.C., Lavonas E.J., Morley P.T. (2020). Part 3: Adult Basic and Advanced Life Support: 2020 American Heart Association Guidelines for Cardiopulmonary Resuscitation and Emergency Cardiovascular Care. Circulation.

[8] Albarran J.W. (2006). Comparison of sequential and simultaneous breathing and pulse check by healthcare professionals during simulated scenarios. Resuscitation Resuscitation.

[9] Yilmaz G., Bol O. (2021). Comparison of femoral and carotid arteries in terms of pulse check in cardiopulmonary resuscitation: A prospective observational study. Resuscitation.

[10] Wyckoff M.H., Singletary E.M., Soar J., Olasveengen T.M., Greif R., Liley H.G., Zideman D., Bhanji F., Andersen L.W., Avis S.R. (2021). 2021 International Consensus on Cardiopulmonary Resuscitation and Emergency Cardiovascular Care Science With Treatment Recommendations: Summary From the Basic Life Support; Advanced Life Support; Neonatal Life Support; Education, Implementation, and Teams; First Aid Task Forces; and the COVID-19 Working Group. Circulation.

[11] Fernandez Castelao E., Boos M., Ringer C., Eich C., Russo S.G. (2015). Effect of CRM team leader training on team performance and leadership behavior in simulated cardiac arrest scenarios: A prospective, randomized, controlled study. BMC Med. Educ..

[12] Perkins G.D., Grasner J.T., Semeraro F., Olasveengen T., Soar J., Lott C., Van de Voorde P., Madar J., Zideman D., Mentzelopoulos S. (2021). European Resuscitation Council Guidelines 2021: Executive summary. Resuscitation.

[13] Merchant R.M., Topjian A.A., Panchal A.R., Cheng A., Aziz K., Berg K.M., Lavonas E.J., Magid D.J. (2020). Part 1: Executive Summary: 2020 American Heart Association Guidelines for Cardiopulmonary Resuscitation and Emergency Cardiovascular Care. Circulation.

[14] Ong M.E.H., Perkins G.D., Cariou A. (2018). Out-of-hospital cardiac arrest: Prehospital management. Lancet.

[15] Sheng J.X., Kan Y., Gai Z., Hao Z. (2021). Public use of external defibrillator in Hangzhou of China. Asian J. Med. Sci..

[16] Panhuyzen-Goedkoop N.M., Wellens H.J., Verbeek A.L.M., Piek J.J., Peters R.J.G. (2021). Immediate Bystander Cardiopulmonary Resuscitation to Sudden Cardiac Arrest During Sports is Associated with Improved Survival—A Video Analysis. Sports Med. Open.

[17] Huang J., Ruse R.B., Walcott G.P., Litovsky S., Bohanan S.J., Gong D.W., Kroll M.W. (2019). Ascending Defibrillation Waveform Significantly Reduces Myocardial Morphological Damage and Injury Current. JACC Clin. Electrophysiol..

[18] Walker R.G., Koster R.W., Sun C., Moffat G., Barger J., Dodson P.P., Chapman F.W. (2009). Defibrillation probability and impedance change between shocks during resuscitation from out-of-hospital cardiac arrest. Resuscitation.

[19] Hess E.P., Russell J.K., Liu P.Y., White R.D. (2008). A high peak current 150-J fixed-energy defibrillation protocol treats recurrent ventricular fibrillation (VF) as effectively as initial VF. Resuscitation.

[20] Ward M.E. (1996). Risk of fires when using defibrillators in an oxygen en-riched atmosphere. Resuscitation.

[21] Theodorou A.A., Gutierrez J.A., Berg R.A. (2003). Fire attributable to a de-fibrillation attempt in a neonate. Pediatrics.

[22] Gibbs W., Eisenberg M., Damon S.K. (1990). Dangers of defibrillation: Injuries to emergency personnel during patient resuscitation. Am. J. Emerg. Med..

[23] Perkins G.D., Kenna C., Ji C., Deakin C.D., Nolan J.P., Quinn T., Fothergill R., Gunson I., Pocock H., Rees N. (2019). The effects of adrenaline in out of hospital cardiac arrest with shockable non-shock-able rhythms: Findings from the, P.A.C.A.; PARAMEDIC-2 randomised controlled, t.r.i.a.l.s. Resuscitation.

[24] Perkins G.D., Kenna C., Ji C., Deakin C.D., Nolan J.P., Quinn T., Scomparin C., Fothergill R., Gunson I., Pocock H. (2020). The influence of time to adrenaline administration in the Paramedic 2 randomised controlled trial. Intensive Care Med..

[25] Callaway C.W., Hostler D., Doshi A.A., Pinchalk M., Roth R.N., Lubin J., Newman D.H., Kelly L.J. (2006). Usefulness of vasopressin administered with epinephrine during out-of-hospital cardiac arrest. Am. J. Cardiol..

[26] Olasveengen T.M., Sunde K., Brunborg C., Thowsen J., Steen P.A., Wik L. (2009). Intravenous drug administration during out-of-hospital cardiac arrest: A randomized trial. JAMA.

[27] Qvigstad E., Kramer-Johansen J., Tomte O., Skalhegg T., Sorensen O., Sunde K., Olasveengen T.M. (2013). Clinical pilot study ofdifferent hand positions during manual chest compressions monitored with capnography. Resuscitation.

[28] Aramendi E., Elola A., Alonso E., Irusta U., Daya M., Russell J.K., Hubner P., Sterz F. (2017). Feasibility of the capnogram to monitor ventilation rate during cardiopulmonary resuscitation. Resuscitation.

[29] Kodali B.S., Urman R.D. (2014). Capnography during cardiopulmonary resuscitation: Current evidence and future directions. J. Emergencies Trauma Shock..

[30] Gutiérrez J.J., Leturiondo M., Ruiz de Gauna S., Ruiz J.M., Azcarate I., González-Otero D.M., Urtusagasti J.F., Russell J.K., Daya M.R. (2021). Assessment of the evolution of end-tidal carbon dioxide within chest compression pauses to detect restoration of spontaneous circulation. PLoS ONE.

[31] Voss S., Rhys M., Coates D., Greenwood R., Nolan J.P., Thomas M., Benger J. (2014). How do paramedics manage the airway during out of hospital cardiac arrest?. Resuscitation.

[32] Piegeler T., Roessler B., Goliasch G., Fischer H., Schlaepfer M., Lang S., Ruetzler K. (2016). Evaluation of six different airway devices regarding regurgitation and pulmonary aspiration during cardio-pulmonary resuscitation (CPR)—A human cadaver pilot study. Resuscitation.

